# Single‐Frequency Birdcage Coils for Deep Tissue Perfluorocarbon Magnetic Resonance Imaging in Mice

**DOI:** 10.1002/nbm.5296

**Published:** 2024-12-08

**Authors:** Sean W. McRae, Francisco M. Martinez, Paula J. Foster, John A. Ronald, Timothy J. Scholl

**Affiliations:** ^1^ Department of Medical Biophysics University of Western Ontario London Ontario Canada; ^2^ Imaging Laboratories, Robarts Research Institute University of Western Ontario London Ontario Canada; ^3^ Lawson Health Research Institute St. Joseph's Health Care London Ontario Canada; ^4^ Department of Physics and Astronomy University of Western Ontario London Ontario Canada; ^5^ Ontario Institute for Cancer Research Toronto Ontario Canada

**Keywords:** ^19^F and ^1^H radiofrequency hardware development, cell tracking, perfluorocarbon imaging, small animal imaging

## Abstract

Fluorine‐19 (^19^F) MRI has become an established tool for in vivo cell tracking following ex vivo or in vivo labelling of various cell types with ^19^F perfluorocarbons (PFCs). Here, we developed and evaluated novel mouse‐specific radiofrequency (RF) hardware for improved dual ^1^H anatomical imaging and deep tissue ^19^F MR detection of PFCs. Three linearly polarized birdcage RF coils were constructed—a dual‐frequency ^1^H/^19^F coil, and a pair of single‐frequency ^1^H and ^19^F coils, designed to be used sequentially. RF coil quality factors (*Q values*), signal homogeneity and sensitivity were benchmarked against a commercially constructed dual‐frequency ^1^H/^19^F surface coil. RF homogeneity was assessed using a phantom designed to mimic PFC localization at depth in a mouse. The single‐frequency birdcage coils (^1^H and ^19^F) displayed more uniform coverage and enhanced signal‐to‐noise ratios (SNRs) compared to both the birdcage and surface dual‐frequency coils for ^19^F detection. Bilateral injection of a perfluoropolyether nanoemulsion into the footpads of female athymic nude mice, resulting in drainage to various lymph nodes and subsequent accumulation in lymph node macrophages, provided a platform to assess differences in SNRs and contrast‐to‐noise ratios (CNR) between both coil configurations as a function of depth and location. The single‐frequency ^1^H coil provided significantly increased CNR in anatomical images (*p* < 0.001) with increased anatomical coverage compared to the dual‐frequency surface coil. The single‐frequency ^19^F birdcage coil offered increased PFC detectability with significantly higher SNR in renal, lumbar, sciatic and popliteal lymph nodes (*p* < 0.01) compared to the dual‐frequency surface coil. Interestingly, the percentage difference between SNR measurements in lymph nodes between the single‐frequency ^19^F coil and the ^1^H/^19^F surface coil had a linear relationship with increasing distance from the surface coil (*R* ^2^ = 0.6352; *p* < 0.0001), indicating a potential disagreement for imaging experiments that rely on ^19^F spin quantification at increasing depth within the mouse using surface RF coils.

## Graphical abstract

This work demonstrates the superior performance of single‐frequency ^1^H and ^19^F birdcage coils for the detection of perfluorocarbons in deep tissue in mice, while maintaining accurate image registration. Our single‐frequency coils were compared against traditional dual‐frequency birdcage and dual‐frequency surface coils, evaluating signal homogeneity, hardware sensitivity and in vivo performance in mice for each coil configuration.

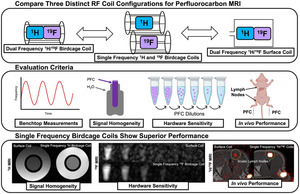

**Abbreviations**
3D‐SPGR: three‐dimensional spoiled gradient recalled echo
bSSFP: balanced steady‐state free precession
CNR: contrast‐to‐noise ratio
NSG: NOD scid gamma
PFC: perfluorocarbon
PFPE: perfluoropolyether
*Q*
: quality factor
RES: reticuloendothelial system
RF: radiofrequency
ROI: region of interest
SNR: signal‐to‐noise ratio

## 1 Introduction

Fluorine‐19 (^19^F) is a useful X‐nucleus for molecular magnetic resonance imaging (MRI) due to its comparable gyromagnetic ratio and relative sensitivity to ^1^H, the ability to directly quantify the number of ^19^F spins and the lack of endogenous background signal [1].^19^F MRI has been explored as a quantitative cell tracking tool for visualizing macrophages [2, 3, 4, 5, 6, 7], dendritic cells [8, 9, 10, 11], T cells [12, 13], natural killer cells [14], β cells [15] and stem cells [16, 17, 18] in small animal models. PFC‐based cell tracking techniques have also been implemented for clinical imaging of labelled dendritic cells [19].

^19^F‐based cell tracking involves either direct ex vivo labelling of cells in culture with a liquid perfluorocarbon (PFC) nanoemulsion followed by adoptive transfer of labelled cells, or one can systemically administer PFCs, which are predominantly taken up by phagocytic cells of the reticuloendothelial system (RES) such as macrophages and monocytes. PFCs consist of fluorine‐rich molecules encased by a lipid coating layer that is internalized and are favourable from a biosafety standpoint as they are sufficiently large (~250 nm) to evade glomerular filtration, removing increased strain on kidneys, instead being cleared by the RES and through exhalation [20, 21]. Additionally, due to the high electronegativity of ^19^F, the C–F bond forms the strongest bond in organic chemistry [22], reducing concerns of free ion circulation.

A significant limitation of ^19^F MRI as a cell tracking modality is sensitivity. While absolute ^19^F and ^1^H detection per atom is similar between the two nuclei, water protons exist in the adult human body at ~60 M concentration, so detection poses little issue. Conversely, MR‐active fluorine is not naturally present in biological subjects (though bones and teeth do contain high concentrations of ^19^F, their incorporation into a solid matrix results in a short T2 sufficiently rendering them undetectable using conventional MR imaging [23]). This results in zero background signal, which is beneficial from a specificity point of view; however, achieving high intracellular concentrations through cell labelling techniques poses a limit on the achievable sensitivity with ^19^F MRI, which is heavily dependent on cell type [19]. For reliable detection with MRI, it is estimated with current techniques that ^19^F concentrations in the millimolar range (10^−3^ M) are required [24, 25]. Therefore, challenges to detection exist, particularly at clinical magnetic field strengths (1.5 or 3 Tesla), such as the efficiency of ex vivo PFC labelling techniques and the localization of the labelled cells to individual voxels [19]. Consequently, long scan times are required to perform signal averaging, oftentimes at lower spatial resolutions, where only localized cellular events that meet the detection limits are reliably observed. Several approaches have been made to enhance the sensitivity of ^19^F MRI through tracer optimization, where research groups have implemented relaxation agents such as paramagnetic Gd(III) ions directly into nanoemulsions in order to shorten the local longitudinal relaxation time of ^19^F spins within labelled cells [26]. Further gains in sensitivity for ^19^F MRI can be made by using ultra‐high field MRI [27] or hyperpolarized MRI [28], though such efforts to improve signal‐to‐noise ratio (SNR) can be both costly and short‐lived, respectively.

At a fixed external magnetic field strength, perhaps the most crucial determinant of sensitivity is the radiofrequency (RF) coil configuration, owing both to the necessity of a uniform B_1_ excitation field within the imaging volume and efficient signal reception. Several groups have explored the use of cryogenic RF probes for ^19^F imaging, which can provide a gain in SNR by reducing thermal noise in the RF coil, though at high field, the sample noise is often dominant, reducing the benefit of RF cooling, depending on coil size [29]. The surface coil is the most fundamental RF configuration for MRI, consisting of a simple planar loop of wire (typically circular or rectangular) that is highly efficient at signal detection when a small‐diameter coil is positioned very close to the subject surface, due to the high filling factor [30]. Multi‐channel phased‐array coils consist of several surface coils used in combination to extend the field of anatomical coverage, though their complex mutual coupling effects and their inhomogeneous B_1_ field have made multi‐channel phased‐array coil configurations less suitable than the more homogenous birdcage coil for mouse imaging [31, 32, 33, 34]. Birdcage coils provide a very uniform B_1_ field, with the potential for an additional increase in SNR when the coil is driven in quadrature. Regardless of coil configuration, ^19^F in vivo imaging experiments require accompanying ^1^H images for anatomical reference to localize the source of PFC signal.

A survey of the literature on small animal PFC MRI reveals that between 2004 and 2024, of the 112 publications that described their coil configurations, 67.0% of authors (*n* = 75) used dual‐tuned volume coils in their experiments, 19.6% (*n* = 22) used dual‐frequency surface coils and only 13.4% (*n* = 15) of authors implemented single‐frequency coils into their imaging experiments. The tendency to favour dual‐frequency coils is likely due to the logistical considerations in image registration and time spent manipulating hardware. While it is true that dual‐frequency coils remove logistical hurdles, the use of active components such as PIN diodes can reduce the SNR due to the added resistance when they are placed in series and are forward biased [35]. Additionally, the non‐negligible intrinsic capacitance of PIN diodes is added to the circuit when they are reversed biased, lowering the overall *Q* and performance of the coil. In contrast, birdcage coils devoted to single‐frequency excitation and detection can be made from simple RLC circuits, increasing the energy storage of the coil, and yielding a higher quality factor (*Q*) at a fixed coil inductance ($Q=\frac{\mathrm{\omega L}}{R}$, where *Q* is the quality factor, ω is the Larmour frequency and *L* and *R* are the inductance and resistance of the circuit, respectively). In this manuscript, we report a ‘back‐to‐basics’ approach to RF hardware implementation for ^19^F molecular MRI in mice, with logistical suggestions for implementing single‐frequency coils while preserving accurate image registration.

## 2 Methods

### 2.1 Coil Design and Construction

**FIGURE 1:**
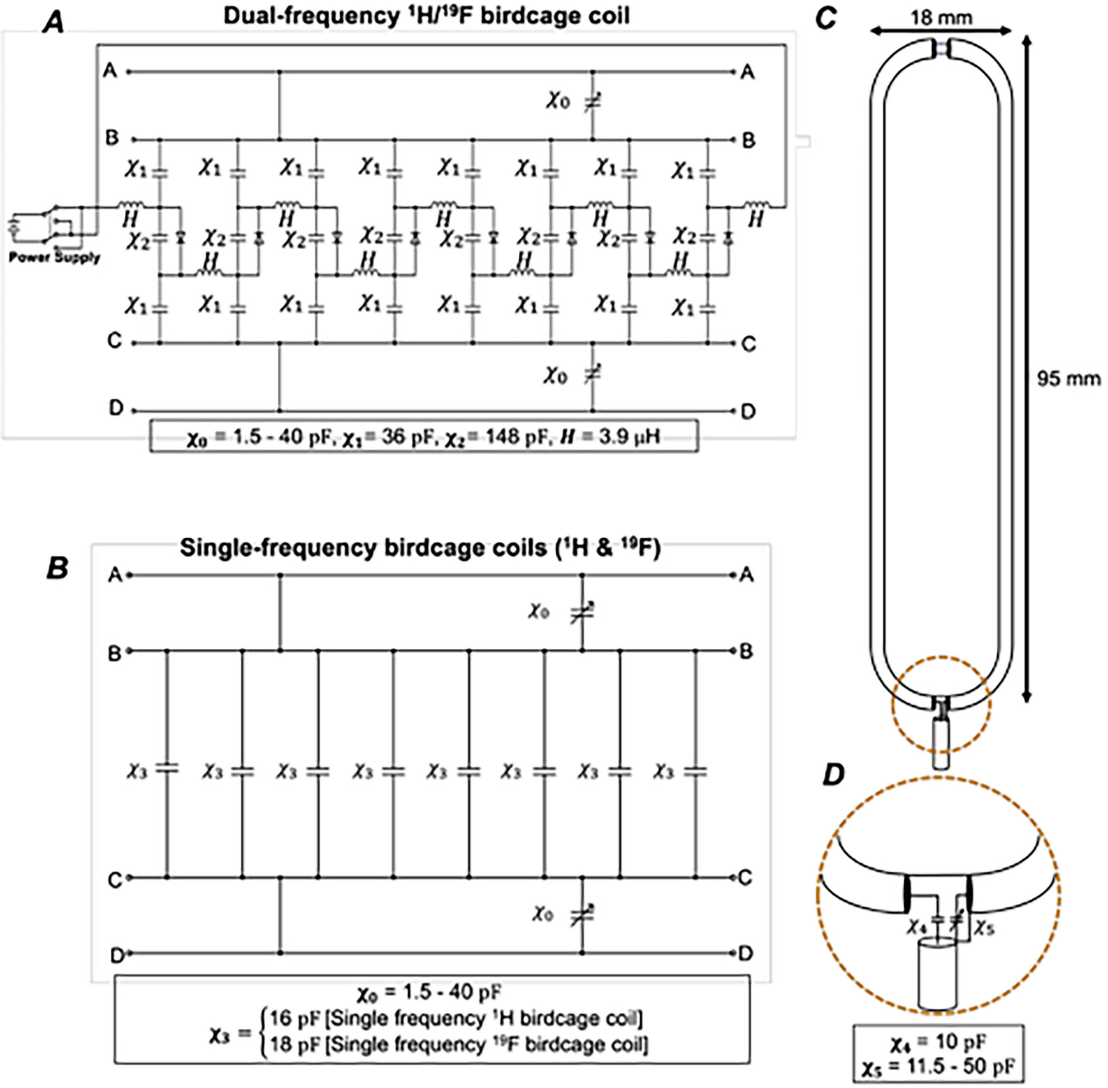
Circuit schematic of the eight‐rung dual‐frequency ^1^H/^19^F birdcage coil (A) and single‐frequency ^1^H and ^19^F birdcage coils (B), where capacitors are denoted by χ and inductors are denoted by H with values listed below each diagram. Schematic of the inductive coupling loop constructed from a single piece of coaxial transmission line (C) and close‐up view of the electrical connections joining the end of the loop (D).

Three unshielded low‐pass custom built mouse birdcage coils; ^1^H single‐frequency, ^19^F single‐frequency and ^1^H/^19^F dual‐frequency were constructed in‐house and designed using Birdcage Builder software (Pennsylvania State University) (Figure 1A,B) [36]. ^1^H and ^19^F single‐frequency coils were constructed using eight copper rungs mounted on an acrylic cylindrical base (dimensions in Table 1). For simplicity of tuning and matching the RF coils to an individual animal at the scanner, we opted to implement linear coupling for these RF birdcage coils. Birdcage coils were designed to provide full mouse coverage for both ^1^H and ^19^F imaging. MR‐compatible ceramic capacitors (Kyosera AVX, formerly American Technical Ceramics) were mounted to the copper rungs to tune the ^1^H and ^19^F coils to achieve 127.728 and 120.05 MHz resonances for imaging at 3 Tesla, respectively, and variable capacitors were added to each copper end ring in parallel to the rung circuit for benchtop frequency adjustments. The switch‐tuned ^1^H/^19^F low‐pass birdcage coil was constructed using capacitors and forward‐biased PIN diodes using forward bias current and reverse voltage to facilitate switching between the 127.728 and 120.15 MHz frequencies. An inductive coupling network employing a 18 mm × 95 mm loop was used for signal reception (Figure 1C) and impedance matching [37, 38]. Inductive coupling circuitry provides good isolation from the resonator that is balanced with respect to the electric fields that lead to signal loss. To further reduce the unwanted influences of electric fields on the imaging experiment, the coupling loop was constructed from a piece of semi‐rigid coaxial transmission line [39]. The outer braiding of the coaxial cable is separated at the peak of the loop to maintain isolation from electric fields but not the magnetic fields. Inductive matching with fixed mutual and variable capacitors was employed to match the impedance of the coil to the RF power supply independent of tuning adjustments. The coupling loop was placed at 8.5 mm from the copper rungs to move the coil into the critical coupling regime and to prevent over‐ or under‐coupling with the resonator [39]. A schematic diagram of a representative birdcage coil (the single‐frequency ^1^H birdcage coil) is shown in Figure S1, with relevant dimensions and photos of the matching circuitry.

**TABLE 1:** Summary of RF coil dimensions.

Coil identity	Frequency (MHz)	Coil diameter (mm)	Rung length (mm)	Rung and end ring width (mm)
^1^H‐birdcage	127.73	31	85	6.5
^19^F‐birdcage	120.15	31	85	6.5
^1^H/^19^F‐birdcage	127.73	31	85	6.5
	120.15			
^1^H/^19^F‐surface coil	127.73	40.3	‐	‐
	120.15			

*Note:* The dimension for the surface coil represents the length of the conductors for the square shape.

An existing commercially constructed surface coil (MR Solutions, Brookfield, WI, USA) designed for local imaging of PFC‐labelled cells in human subjects as well as mice [13, 40] was used in this study to act as a standard.

### 2.2 Design and Construction of MR‐Compatible Stationary Animal Cradle

**FIGURE 2:**
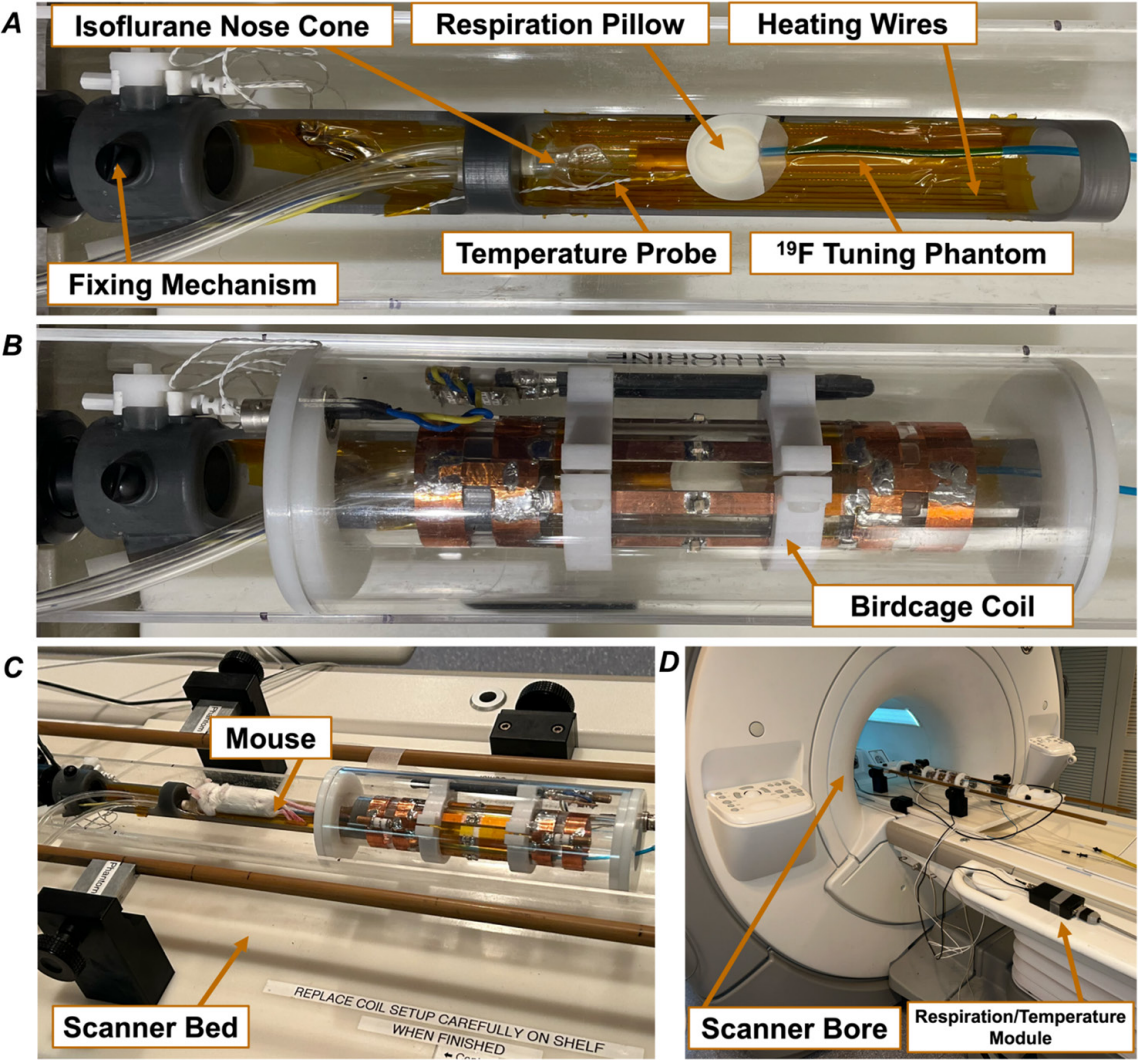
Photos of the in vivo imaging setup on the General Electric Healthcare Discovery MR750 3.0T™ clinical MR scanner. Top‐down view of the stationary animal cradle before (A) and after (B) coil positioning with denoted locations of the fixing mechanism that maintains the mouse's position relative to the scanner bed during coil switching, isoflurane nose cone, temperature probe, respiration pillow, heating wires and ^19^F tuning phantom indicated. Set up with mouse on the animal cradle (C), and setup on the scanner bed for scale (D).

The use of individual single‐frequency ^1^H and ^19^F birdcage coils for in vivo animal imaging motivated the construction of a system that could keep the animal stationary and under anaesthesia while switching between coils during imaging sessions. Our approach was to construct an animal cradle that was fixed to the scanner bed, which would allow the exchange of single‐frequency ^1^H and ^19^F birdcage coils without moving the animal in order to preserve image co‐registration (Figure 2A,B). The animal cradle incorporated provisions for isoflurane anaesthesia and scavenging, resistive heating of the animal bed, and animal temperature and breathing sensors (Figure 1C). The cradle also contained a fiducial NMR tube (4 mm inner diameter, 10 cm in length) filled with 875 μL of PFC of known fluorine density mounted to the cradle to provide a source of ^19^F for centre frequency tuning and serve as a standard reference throughout imaging. Images of a mouse on the scanner bed are shown to demonstrate the scale of the imaging experiment in the General Electric Healthcare Discovery MR750 3.0T™ clinical MR scanner (Figure 2C,D).

### 2.3 *Q* Measurements

*Q values* were measured using a E5061B ENA Series Network Analyser (Agilent Technologies, Santa Clara, CA, USA), using the 3 dB method [41]. S21 measurements were conducted for each coil (the three constructed birdcage coils as well as the reference MR Solutions surface coil), both unloaded and loaded with an ~30 mL Gd‐doped water phantom, after tuning and matching to the load, or the empty coil. Using measured loaded and unloaded *Q* factors $\left(Q_{L}\text{and}\;Q_{\mathrm{UL}},\text{respectively}\right)$, the theoretical percentage of a coil's maximum SNR that is achievable was calculated using the following equation: ($\sqrt{1-Q_{L}/Q_{\mathrm{UL}}}$) [42]. Additionally, an estimation of the source of noise dominance within the RF coil can be made by taking the ratio of the *Q* for the unloaded coil to the *Q* of the loaded coil, where a ratio >2 indicates sample‐noise dominance and a ratio <2 indicates coil‐noise dominance [30].

### 2.4 PFC Agent

The PFC agents Celsense (CS‐1000) and V‐Sense (VS‐1000) were obtained from Celsense Inc. (Pittsburgh, PA, USA) [43]. CS‐1000 is comprised of linear perfluoropolyether (PFPE) polymers bound in a nanoemulsion, with an average diameter of 170 nm at a concentration of 100 mg of ^19^F per mL of agent [44]. V‐Sense contains an average droplet size of 145 nm, containing 20% volume fraction of PFC in a buffered solution to facilitate uptake into macrophages in vivo.

### 2.5 Coil Homogeneity Measurements

A standard phantom was constructed to facilitate both ^1^H and ^19^F imaging using concentric plastic tubes (diameter 23 mm and length 55 mm). The outer portion of the phantom contained Milli‐Q deionized water doped with gadopentetic acid (Gd‐DTPA) (Magnevist, Bayer, Leverkusen, Germany), while the inner tube (dimensions 46 mm × 12 mm) contained ~2 mL of the ^19^F nanoemulsion Celsense (Celsense Inc., Pittsburgh, PA, USA). Coronal images of the dual‐frequency phantom were obtained for SNR and homogeneity analysis on a clinical 3‐Tesla MRI scanner (General Electric Healthcare Discovery MR750, Milwaukee Wisconsin, USA). Proton images were acquired using a spoiled gradient echo sequence with FOV 7.7 cm × 2.7 cm, TR/TE = 10.5 ms/3.62 ms, flip angle = 10°, 4 signal averages and matrix size 256 × 256 (scan time 6 min). Fluorine images were obtained using a balanced steady‐state free precession (bSSFP) sequence with FOV 10 cm × 3 cm, TR/TE = 7.3 ms/2.732 ms, flip angle = 72°, 10 signal averages and a matrix size of 100 × 100 (scan time 10 min). To assess the signal homogeneity throughout a coil's imaging volume, SNR was calculated for each coronal imaging slice comprising the phantom and plotted as a function of depth. The SNR‐depth calculations and plots were generated using a custom MATLAB script (R2021a, MathWorks, Natick, Massachusetts, USA). To further evaluate RF homogeneity through the volume of the coils, B_1_^+^ maps were acquired using the Bloch–Siegert Shift method [45] with a flip angle of 30° (Figure S2). Maps were generated for the single‐frequency ^1^H birdcage coil (as a representative coil, since homogeneity should be similar for the other birdcage coils constructed with the same components and with the same dimensions) and the dual‐frequency ^1^H/^19^F surface coil (proton frequency).

### 2.6 In Vitro ^19^F Detection Limit Measurements

To compare imaging sensitivity across the ^19^F‐compatible coils, we created standard ^19^F samples with known numbers of ^19^F spins and compared achievable SNR at a standard scan time of 30 min. Samples were prepared by making a sample containing 2.63 μL of CS‐1000 in 57.37 μL and performing 1:2 dilutions in a constant volume of 60 μL per sample for a total of six vials (ranging from 5.1 × 10^16^ to 8.4 × 10^14 19^F spins/μL). The concentration of ^19^F spins were measured and confirmed using NMR. In addition to the samples, a 0.1% trifluoroacetic acid was included as a reference and deuterated water for ^19^F spectra shimming. ^19^F NMR spectra were acquired at 25°C using a Bruker Neo 600 NMR spectrometer equipped with a Bruker 5 mm HX iProbe. For each spectrum, a total of 64 scans were summed using a 45° tip angle, a 6.5 s recycle delay, a 4.72 s acquisition time and a spectral width from −50 to −100 ppm. The raw NMR data were processed using Mestrelab's Mnova NMR software package. FIDs were apodized with 1 Hz line broadening and zero‐filled two times before Fourier Transform. Baseline correction was performed using a 4th‐order polynomial fit before integration. NMR spectra are included in Figure S3.

Phantoms were then aligned in a single row along the bore when imaging with birdcage coils, and phantoms were aligned in two rows of three and placed within the space of the surface coil loop. Fluorine images were obtained as above. The imaging time was fixed at 30 min for each coil; given the differences in the field of view between the surface coil and the birdcage coils (6 cm × 3 cm and 10 cm × 3 cm, respectively), this resulted in 144 signal averages for the surface coil and 120 signal averages for the birdcage coils. A series of three images were acquired using the same phantoms, spaced 1 day apart (*n* = 3). Regions of interest (ROIs) were drawn on the ^19^F images based on registration with the proton image to compare SNR differences between coils for each vial.

### 2.7 In Vivo ^1^H/^19^F MRI

All in vivo experiments were completed in accordance with our animal use protocol (AUP 2022‐190), and Western University's animal use guidelines. V‐Sense (50 μL) was injected bilaterally into the footpads of athymic nude mice (*n* = 3). Animals were anaesthetized using isoflurane gas (3% induction, 2% maintenance in oxygen). Anatomical T_1_‐weighted images were acquired using a 3D‐spoiled gradient recalled acquisition in the steady state (3D‐SPGR) with the following image parameters: FOV = 12.0 cm, TR = 14.7 ms, TE = 2.456 ms, rBW = 62.5 kHz, matrix size = 400 × 400, flip angle = 60°, NEX = 1, voxel size = 300 μm^3^ isotropic, scan time = 9 min. ^19^F images were obtained using a bSSFP sequence, FOV 12 cm × 3 cm, TR/TE = 7.3 ms/2.732 ms, flip angle = 72°, 120 signal averages, matrix size of 100 × 100, voxel size 1 mm and scan time 30 min. When the single‐frequency birdcage coils were used, the ^1^H images were first acquired before switching to the ^19^F coil. The switching process took <60 s, with additional time (less than 3 min) added to re‐tune and match the RF coil before acquiring ^19^F images. Mice were scanned 1‐, 8‐, and 24‐days post injection of PFC tracer. Additional photos of the in vivo imaging setup in the scanner are supplied in Figure S4.

Image co‐registration between anatomical images and PFC maps was confirmed for the birdcage coil images through alignment of the ^19^F reference phantom, which provided low but useable ^1^H signal on T_1_‐weighted images due to the long T_1_ of the PFC solution. Some images required small vertical transformation for full registration, which could be due to off‐resonance effects, but no image sets required any more complex image registration steps (rotational, deformable, etc.).

ROIs were created on ^1^H anatomical images within a central slice of each lymph node and applied to ^19^F images for assessment of SNR of PFC detection. Drawing ROIs based on the ^1^H images helped reduce bias in reporting ^19^F SNR values, while allowing an estimate of SNR to be made for lymph nodes that either contained no PFC or contained not enough to be confidently detected.

To further investigate the influence of depth on the performance of the surface coil for detection of PFCs in deep tissue, sagittal MR images were analysed to determine the distance from the surface coil to the lymph node in millimeters (estimated by making distance measurements on sagittal MR images, where the start of the measurement point was taken to be the edge of the phase encode direction, along that negative y‐axis where the surface coil was positioned anteriorly to the mouse's abdomen). Percentage differences between lymph node ^19^F SNR between the two coils was calculated following the equation below:

$$\text{Percentage Difference}=100\times \frac{|{\mathrm{SNR}}_{\text{Birdcage Coil}}-{\mathrm{SNR}}_{\text{Surface Coil}}|}{\frac{1}{2}\left({\mathrm{SNR}}_{\text{Birdcage Coil}}+{\mathrm{SNR}}_{\text{Surface Coil}}\right)}$$

Finally, to investigate the presence of banding artifacts in in vivo ^19^F images, 200 μL of V‐Sense was injected intravenously into a healthy female NOD scid gamma (NSG) mouse (*n* = 1) and scanned with the same bSSFP sequence described above. NSG mice lack mature lymph nodes and functional macrophages [46] and therefore yield more tracer uptake in the liver from which to assess banding in a large organ. In this experiment, all three coil configurations were used for scanning (single‐frequency ^1^H and ^19^F birdcage coils, dual‐frequency ^1^H/^19^F birdcage coil and the dual‐frequency ^1^H/^19^F surface coil). Banding was assessed by generating line profiles in the superior/inferior direction on coronal ^19^F images, plotting pixel‐by‐pixel SNR. Calculations were performed using a custom MATLAB script (R2021a, MathWorks, Natick, Massachusetts, USA).

### 2.8 Statistics

A two‐way ANOVA with Tukey multiple comparisons was used to determine significance in SNR differences between coils for each vial, and a Šídák multiple comparisons test was used to determine statistical difference in SNR measured within lymph nodes between the single‐frequency birdcage coil and the ^1^H/^19^F surface coil.

## 3 Results

### 3.1 *Q* Measurements

*Q* factor measurements for each coil are listed in Table 2. The ideal RF coil will have a very high *Q* when the coil is unloaded, with a substantial drop when the coil is loaded [42]. The theoretical percentage of maximum SNR achievable indicates the expected percentage of a coil's maximum SNR compared to a perfect, lossless coil [47].

**TABLE 2:** Quality factor measurements for each of the three custom built birdcage coils and the MR Solutions dual‐frequency surface coil.

Coil identity	$f_{0}$ (MHz)	Quality factor (*Q*)			Noise dominance	$\sqrt{1-Q_{L}/Q_{\mathrm{UL}}}$
		*Q*_*UL*_ (unloaded)	*Q*_*L*_ (loaded)	*Q*_*UL*_/*Q*_*L*_		
^1^H‐birdcage	127.73	330	140	2.36	Sample	76%
^19^F‐birdcage	120.15	340	167	2.03	Sample	71%
^1^H/^19^F‐birdcage	127.73	275	155	1.77	Coil	66%
	120.15	95	70	1.36	Coil	51%
^1^H/^19^F‐surface coil	127.73	104	99	1.05	Coil	22%
	120.15	123	112	1.10	Coil	30%

*Note:* Measurements were made before and after loading with an ~30 mL Gd‐doped water phantom. The final column estimates the theoretical proportion of a coil's maximum SNR that is expected based on the measured *Q* factors.

Based on *Q* factor measurements, both single‐frequency birdcage coils are sample‐noise dominated whereas the dual‐frequency birdcage and surface coils are coil‐noise dominated. Additionally, the single‐frequency birdcage coils are predicted to achieve a higher percentage of their maximum SNR by a factor of 2.4 and 1.4 for ^19^F imaging and a factor of 3.5 and 1.1 for ^1^H imaging compared to the dual‐frequency surface coil and dual‐frequency coils, respectively.

### 3.2 Increased SNRs and Signal Homogeneity

**FIGURE 3:**
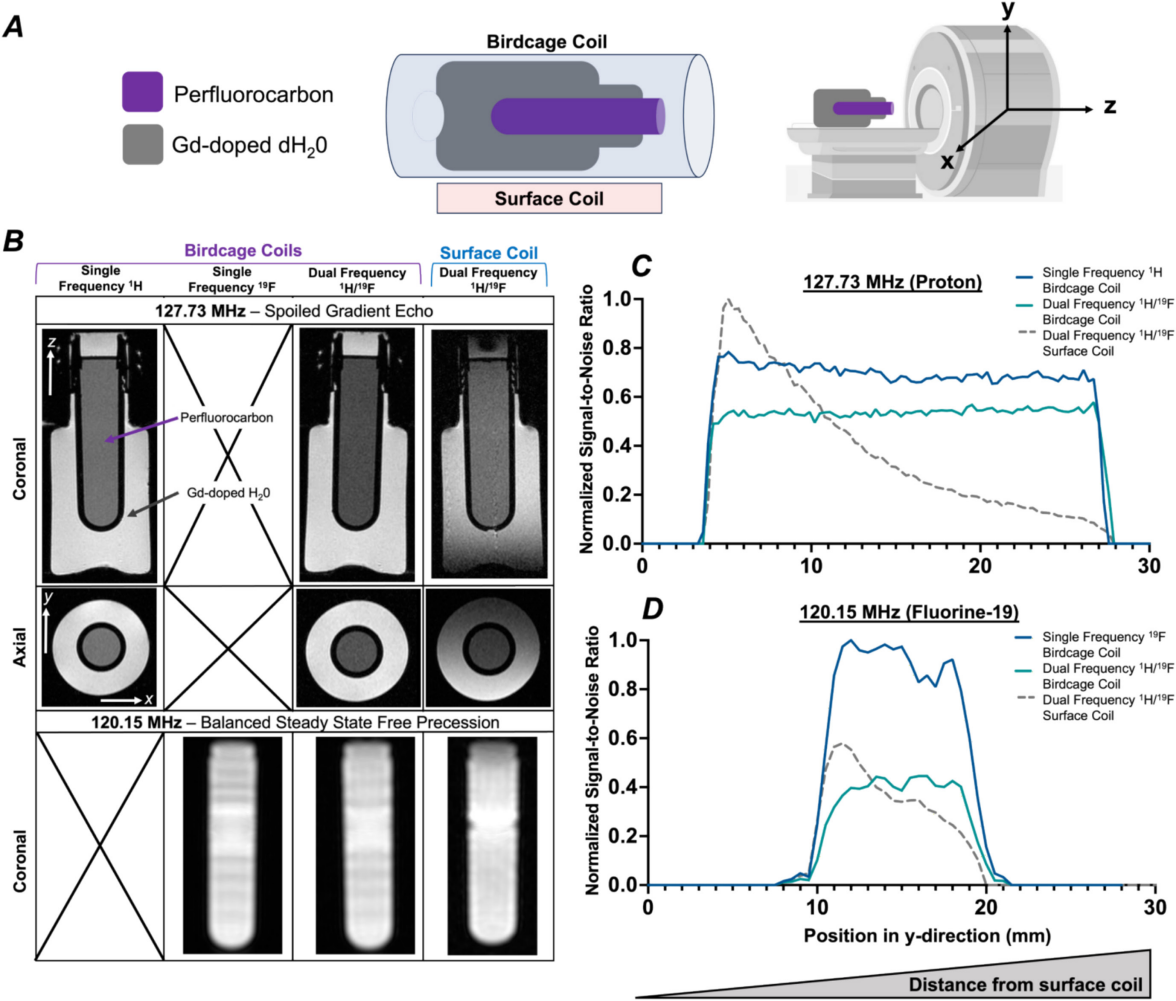
Phantom imaging evaluating coil homogeneity between single‐frequency birdcage coils, and the ^1^H/^19^F birdcage and the ^1^H/^19^F surface coil (A). Surface coil positioned along the x‐axis as seen in the axial images. Representative proton images (coronal and axial) and fluorine‐19 images (coronal) (B). SNR calculation as a function of depth for proton (C) and fluorine‐19 images (D).

The phantom containing both ^1^H and ^19^F nuclei was oriented along the bore of the scanner (along the z‐axis), and coronal images were acquired for both frequencies for all RF coils (Figure 3A). Axial ^1^H images demonstrated the expected signal drop‐off effect with increasing distance from the surface coil. Coil homogeneity for both imaging frequencies were evaluated through a slice‐by‐slice measure of SNR within a central region of the phantom. SNR‐depth curves for both ^1^H and ^19^F are shown below (Figure 3B,C, respectively), normalized to the maximum SNR for each frequency.

Proton SNR‐depth curves reveal higher signal strength for the surface coil over the birdcage coils, when closest to the phantom. However, the SNR received from the surface coil exhibited exponentially decreasing sensitivity (*R* ^2^ = 0.9939) at depths past the maximum signal depth of 5.1 mm. The analogous dual‐tuned birdcage coil and the single‐frequency ^1^H birdcage coils exhibited highest sensitivities at depths of 5.4 and 5.1 mm, respectively, with peak SNRs of 55.2% and 78.4% relative to the surface coil. Despite the slightly lower relative proton sensitivity of the birdcage coils at depths less than 8 mm compared with the surface coil, they did provide enhanced homogeneity across the volume of the phantom relative to the surface coil.

Fluorine SNR‐depth curves displayed a similar appearance to proton results, with the surface coil providing its highest signal at depths closest to the phantom at 11.5 mm followed by a one phase decay at greater depths (*R* ^2^ = 0.9389). However, in this case, the SNR_MAX_ of the surface coil achieved only 57.9% of the maximum SNR for the single‐frequency birdcage coil, with the ^1^H/^19^F birdcage coil displaying the lowest peak SNR at only 44.6% of the maximum SNR of the single‐frequency ^19^F birdcage coil.

### 3.3 ^19^F Detection Limit Measurements

**FIGURE 4:**
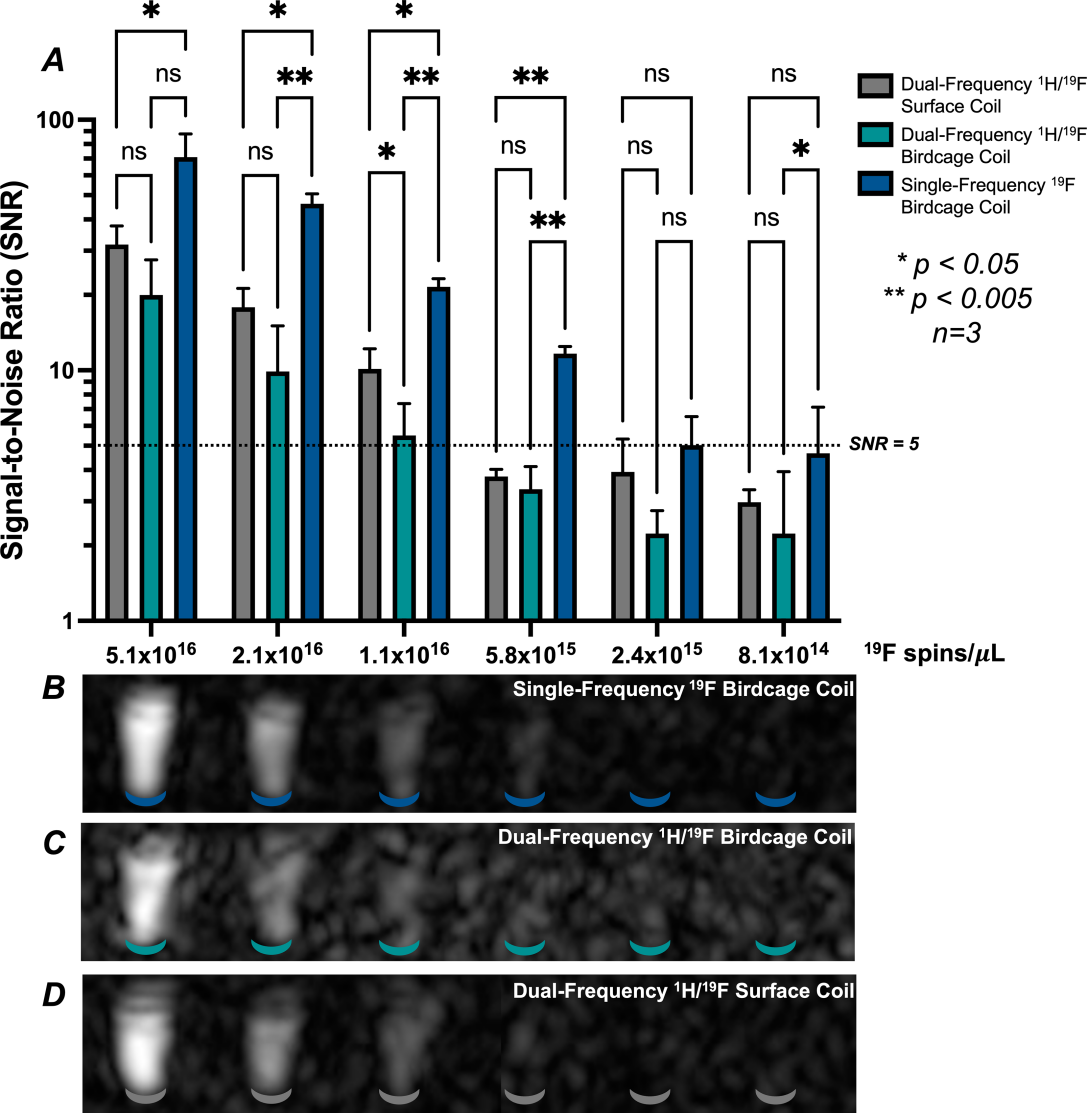
Summarized analysis of fluorine‐19 images of CS‐1000 sensitivity phantoms (A) with representative sagittal MR imaging from the ^19^F single‐frequency birdcage coil (B), dual‐frequency birdcage coil (C) and dual‐frequency surface coil (D).

Differences in ^19^F detection limits between RF coils was directly assessed using phantoms containing known numbers of ^19^F spins, removing the influence of depth (Figure 4). Samples were placed vertically along the bore of the birdcage coils, and within the loop of the surface coil for maximum signal reception.

Analysis of SNR in coronal slices within each vial demonstrated significantly superior sensitivity for the single‐frequency birdcage coil over the surface coil in the first four vials (*p* < 0.04), and over the dual‐tuned birdcage coil in Vials 2–4 (*p* < 0.005; Figure 4A). The Rose criterion was used to define the minimum acceptable SNR for confident detection (SNR > 5). An SNR greater than 5 was met in Vials 1–5 for the single‐frequency ^19^F birdcage coil (Figure 4B), and only in Vials 1–3 for the dual‐frequency birdcage coil (Figure 4C) and dual‐frequency surface coil (Figure 4D).

### 3.4 In Vivo Detection of ^19^F in Deep Tissue

**FIGURE 5:**
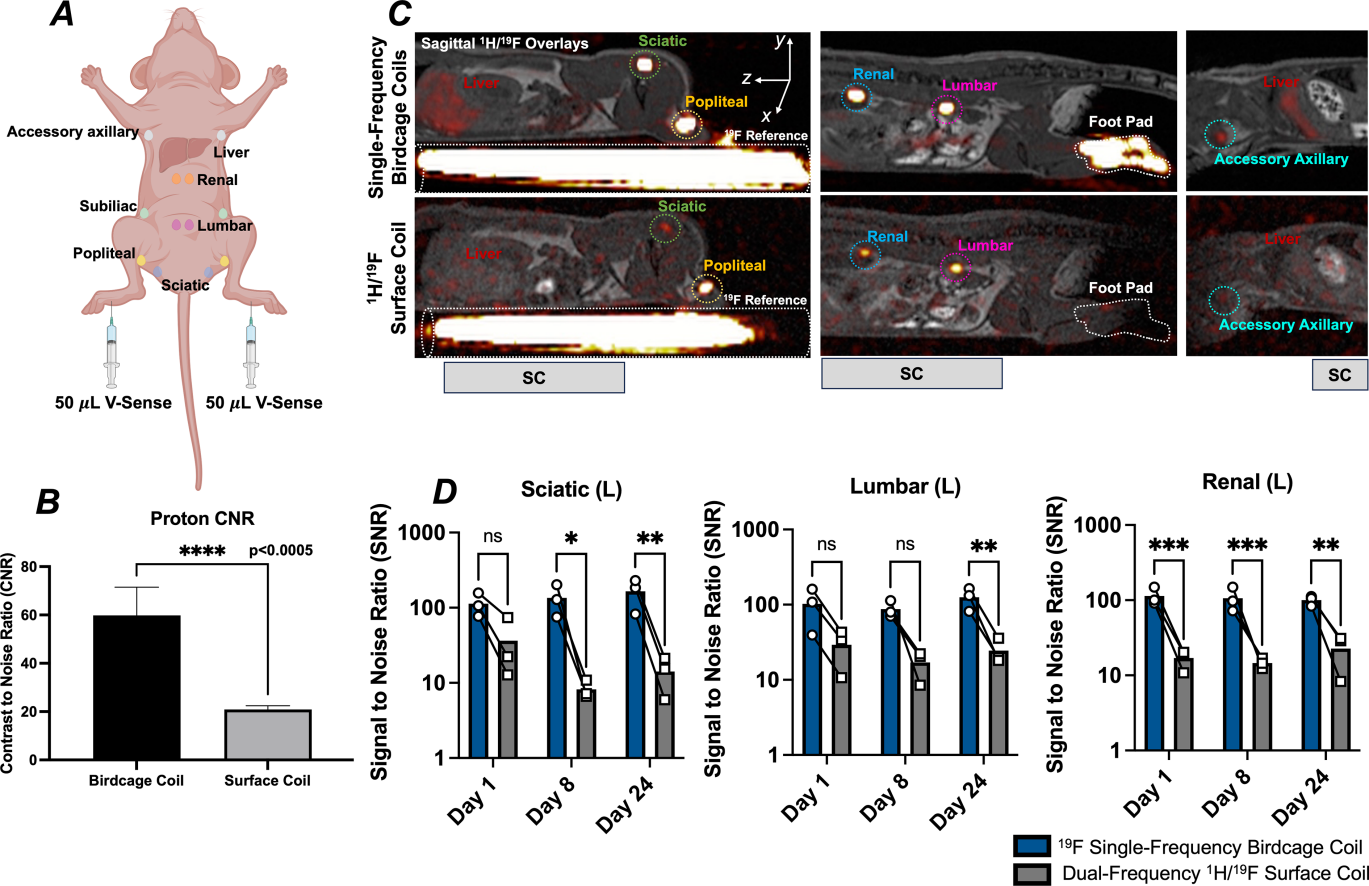
Expected biodistribution of V‐Sense upon bilateral injection into footpads (A). Proton CNR measured between muscle and fat for the single‐frequency ^1^H birdcage coil and the dual‐tuned surface coil (B). Representative slice‐ and window/level‐matched ^1^H/^19^F sagittal overlays of a selection of lymph nodes assessed in SNR analysis with the approximate position of the surface coil denoted by a grey bar on the anterior side of the mouse (C). Summarized ^19^F SNR differences between ^19^F single‐frequency coil and the dual‐frequency surface coil with connecting lines indicating SNR differences within individual lymph nodes between the two ^19^F coils (* *p* < 0.05, ** *p* < 0.01, and *** *p* < 0.0005) (D).

Injection of PFC into the footpads of nude mice results in drainage to the nearby popliteal lymph node, with subsequent drainage to the iliac and renal lymph nodes, or directly from the footpads to the inguinal/lumbar and axillary lymph nodes with additional drainage to sciatic lymph nodes [48] (Figure 5A), with clearance through the Kupffer cells in the liver. When V‐Sense drains through the lymphatic vasculature, nearby phagocytic lymph node macrophages can internalize the PFC and produce signal in MR imaging for extended timeframes [49, 50]. The presence of lymph nodes at various depths within the mouse provided an in vivo basis for comparing coil performance in tissues at varying depth. Based on their superior performance compared to the dual‐frequency birdcage coil in the phantom experiments, only the single‐frequency birdcage coils and dual‐frequency surface coil were used for in vivo imaging.

Anatomical proton maps were analysed by calculating CNR between the hind limb muscle and subdermal adipose tissue. CNR measurements using the ^1^H birdcage coil were significantly higher than with the dual‐frequency surface coil (*p* < 0.0005; Figure 5B). Additionally, birdcage coils provided improved anatomical coverage, allowing the head and the base of the tail of the mouse to be captured within the field of view. A representative image set from the co‐registered ^1^H and ^19^F birdcage coil images are shown for a mouse 8 days after PFC injection (Figure 5C). Changes within lymph node SNR between coils are indicated by connecting lines at each time point (Figure 5D), revealing statistically significant differences in the left sciatic, left lumbar and left renal lymph nodes at the indicated time points. Comparisons of the SNR within the remaining deep lymph nodes over the three imaging time points is summarized in Figure S5. Briefly, though the ^19^F‐single‐frequency RF coil provided enhanced sensitivity compared to the ^1^H/^19^F surface coil, not all mice exhibited PFC signal in all lymph nodes, reducing significance in the accessory axillary and subiliac lymph nodes.

**FIGURE 6:**
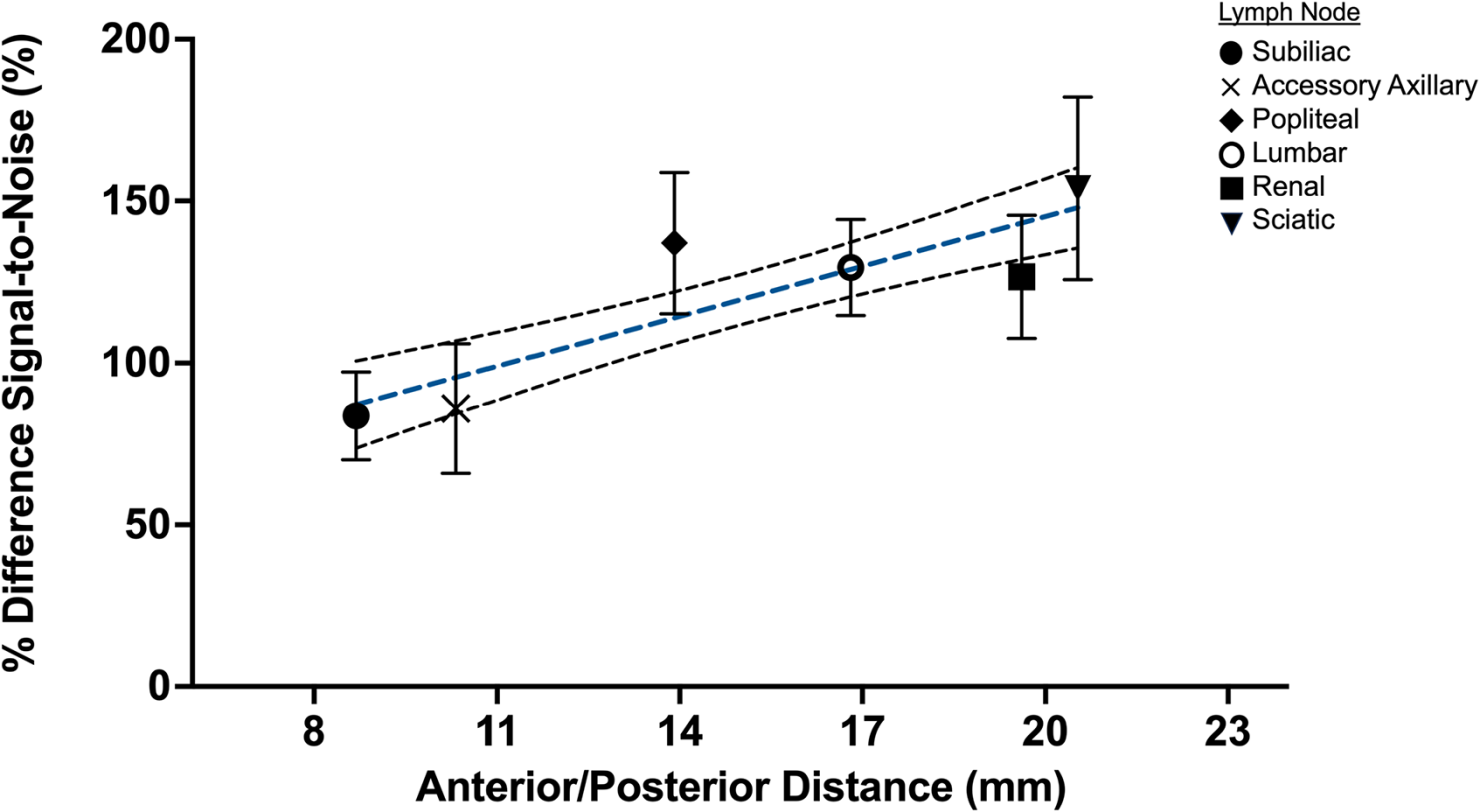
Relationship between depth and percentage difference in SNR between the surface coil and single‐frequency birdcage coils with linear regression fit shown with all six lymph nodes (including the popliteal lymph nodes). The curved black lines indicate the 95% confidence interval of the fit.

Average percentage difference in SNR between mice across time for various lymph nodes was plotted as a function of approximate distance from the surface coil measured from sagittal MR images (Figure 6). A linear regression analysis with a goodness‐of‐fit test revealed an *R* ^2^ fit coefficient of 0.5097. The removal of the popliteal lymph node signal, which was highly contaminated by residual PFC in the footpads after injection in some animals, improves the fit to an *R* ^2^ of 0.6352, indicating good correlation between distance and percentage difference between SNR measurements.

In the mouse that received a systemic injection of 200 μL of V‐Sense, the presence of banding artifacts is most pronounced in the ^19^F image acquired using the ^1^H/^19^F dual‐frequency surface coil. SNR line profiles reveal the presence of a series of three local minima at 34, 48 and 63 mm along the line of measurement with signal drops of 24%, 26% and 43% from their local maxima, respectively. While the images acquired with the birdcage coils were also susceptible to banding (see short yellow arrows), the presence of local minima/maxima was less striking in the single‐frequency coil where local minima were present at 41, 50 and 57 mm, though SNR drops were lower at 2%, 3% and 6%, respectively. Lastly, the dual‐frequency birdcage coil presented with two local minima at 43 and 57 mm, with drops in SNR of 9% and 25% relative to their local maxima, respectively (Figure S6).

## 4 Discussion

The choice of RF hardware can have significant impact on in vivo MR imaging experiment and must be given careful thought, particularly when low concentrations of nuclei are located at depth. Here, we demonstrated the sensitivity and homogeneity advantages of using easily exchangeable single‐frequency ^1^H and ^19^F birdcage coils with whole‐mouse coverage over both a whole‐mouse dual‐frequency birdcage coil and previously utilized partial‐mouse dual‐frequency surface coil.

In many ^19^F cell tracking studies, a priori knowledge of the location of labelled cells would be necessary to properly place a surface coil to maximize sensitivity. Thus, a homogenous whole‐mouse coil with equivalent or improved sensitivity and improved quantitation capabilities at any depth would be highly beneficial. The in vivo lymph node imaging study presented here served as a platform for directly comparing the performance of two distinct RF coil configurations for PFC detection in deep tissue. Though the single‐frequency ^19^F birdcage coil provided higher SNR compared to the surface coil on average within each lymph node, differences were not always statistically significant. The SNR of high‐*Q* coils can be highly variable depending on the quality of the tuning and matching achieved with a loaded coil on the scanner bed, despite our best efforts to optimize the coil before imaging. Additionally, the limited anatomical coverage of the surface coil introduced another source of variability in the measurement of signal within the accessory axillary and popliteal lymph nodes that lie the most superior and inferior to the mouse, respectively, as the position of the surface coil relative to the mouse could vary from day to day. Lastly, biological differences in PFC uptake between mice and the quality of footpad injection will also introduce sources of variation within the SNR measurements made here. Though our results show that the highest difference in ^19^F SNR measurements between single‐frequency birdcage coils and dual‐frequency surface coil was observed in the sciatic lymph nodes of mice, it is the deep‐set lumbar lymph node (located at approximately half of the total depth of the mouse) that would benefit the most from imaging with single‐frequency birdcage coils; as for these lymph nodes, there is no way of moving the coil closer to the target, and flipping the mouse from prone to supine position is not an option. The discrepancy in signal reception between the surface coil and single‐frequency birdcage coils as depicted in Figure 6 would lead to a significant systematic error in cell number for cell tracking at the deepest point in the animal, where cells are labelled with PFCs in culture and cell loading is measured using NMR to determine in vivo cell number.

The balanced steady‐state free precession pulse sequence used in this study for ^19^F imaging is a well‐documented and clinically implemented pulse sequence for ^1^H imaging that provides exceptional SNR by the balancing of gradients across a TR to generate complex T_2_/T_1_ contrast [51]. However, this sequence is extremely sensitive to magnetic field inhomogeneities, which become more pronounced at high field strength and at long TR for high resolution scanning, even with increased number of phase cycles used [52]. The presence of persistent banding artifacts due to an inhomogeneous B_0_ field is exacerbated by the inhomogeneous B_1_ field that the surface coil provides at depth. The homogenous B_1_ field generated by the birdcage coils can provide improved quantification through the reduction of banding artifacts.

The RF coils presented here were specifically tailored for imaging applications in mice, but the principle of using single‐frequency coils alongside a method for proper coil switching can be extended to both larger animals, and to image more than two nuclei. As always, the filling factor predicts that a coil that fits close to the imaging subject will yield higher SNR by increasing the sample resistance, so extending this system to larger animals is possible as long as the coil dimensions are scaled accordingly. The birdcage coils constructed here were designed to be linearly polarized, but further gains in sensitivity for both frequencies are possible when coils are driven in quadrature [53, 54]. Our experience constructing murine RF coils suggests that for small‐bore birdcage coils, the expected gain in SNR from driving the coil in quadrature may not be seen in practice. This is likely due to the limited isolation between quadrature channels, which is often the situation for small RF coils, suggesting further gains in SNR could be achieved for imaging larger animals.

One limitation of the coil construction presented here was the need for a large PFC‐containing sample to be permanently fixed to the animal bed throughout imaging. In other MR experiments that use planar surface coils, a large PFC sample can be placed temporarily within the coil FOV for frequency and flip angle calibration and removed before imaging, and smaller ^19^F reference vials with known spin numbers can be placed under the imaging subject for later quantification. In the design presented here, a single tube was used for both frequency/flip angle calibration and reference (though a detailed quantification was not presented in this study). Consequently, since the ^19^F sample needed to be close to the centre of the birdcage coil's volume for accurate calibration, the vial was positioned very close to the mouse. The high number of ^19^F spins contained within the reference sample and the lack of comparable biological ^19^F signal lead to a high contrast interface and truncation of the Fourier series, which leads to a series of over and undershoot oscillations and false widening of the sample edges [55]. These oscillating artifacts lead to contamination of signal within nearby lymph nodes, though this effect was not specific to the birdcage coils as the same reference sample was used with the surface coil. In a future iteration of this, dual‐coil system will incorporate a large removable ^19^F vial for frequency and flip angle calibration, and smaller, lower concentration reference samples will be fixed at appropriate positions within the coil volume. However, this strategy may prove challenging as there is limited space within the coil volume due to the necessary animal monitoring equipment.

Though this report primarily outlined improvements in ^19^F signal acquisition, there were numerous advantages seen for ^1^H imaging with the single‐frequency ^1^H birdcage coil, namely, improved anatomical coverage and statistically increased CNR. The improved CNR from a devoted ^1^H birdcage coil would allow for improved detectability of cell populations in vivo using proton‐based cell tracking tools [56, 57, 58, 59] opening the door for highly sensitive dual ^1^H/^19^F cell tracking studies. In future studies, we will apply this new hardware to perform dual cell tracking studies that can combine the gains in sensitivity that have been achieved for both frequencies.

## 5 Conclusions

We posited that single‐frequency birdcage coils retain higher absolute sensitivity compared to their dual‐frequency counterparts due to their simplistic RLC circuits, effectively lowering the overall resistance. Our data support the use of single‐frequency RF coils in the birdcage configuration to allow for improved coverage and homogeneity across the whole animal due to the uniform B_1_ excitation field generated within its volume. This will be essential for studies without a priori knowledge of signal location and becomes particularly important in cell tracking studies where the biodistribution of cells can be unknown from the time of injection to endpoint, as well as for monitoring off‐target effects during studies involving targeted therapeutics.

## Author Contributions

T.J.S and J.A.R are co‐senior authors.

## Supporting information


**Figure S1.** Birdcage coil schematic diagram with relevant dimensions (A), labelled diagram of inductive coupling loop (B), close‐up photos of the matching circuitry front (C) and back (D).
**Figure S2.** B_1_^+^ maps acquired for ^1^H excitation with the single‐frequency ^1^H birdcage coil and the dual‐frequency ^1^H/^19^F surface coil using the Bloch–Siegert Shift method with a flip angle of 30°. The 50 mL 0.5% agarose phantom doped with 200 μL of Gd‐DTPA, axial (B) and coronal (C) slices of the B_1_^+^ map and corresponding magnitude image for the single‐frequency ^1^H birdcage coil, and axial (D) and sagittal (E) slices of the B_1_^+^ map and corresponding magnitude image for the dual‐frequency ^1^H/^19^F surface coil.
**Figure S3.** NMR analysis of six samples of decreasing ^19^F concentrations used for in vitro sensitivity measurements. Large TFA reference peak and CS‐1000 sample resonance peaks are labelled.
**Figure S4.** Additional photos of in vivo imaging setup on the General Electric Healthcare Discovery MR750 3.0T™ clinical MR scanner. View of birdcage coil and animal cradle on scanner bed during experiment set up (A), imaging set up inside the bore of the MR scanner (B), animal heating and temperature control module held within the control room (C).
**Figure S5.** Full survey of ^19^F SNR measurements in accessory axillary, sciatic, popliteal, renal, subiliac and lumbar lymph nodes, showing changes in individual SNR measurements due to differences in coil performance.
**Figure S6.** Coronal ^19^F MR images of PFC localization in the livers of mice, showing differences in bSSFP banding from each of the three ^19^F compatible coils (dual‐frequency surface coil, dual‐frequency birdcage coil and single‐frequency birdcage coil). Line profiles showing SNR as a function of vertical position from analysis of a vertical cut (indicated by the dotted yellow line).

## Data Availability

The data that support the findings of this study are available from the corresponding author upon reasonable request.
