# Supplementary material for: Single‐Frequency Birdcage Coils for Deep Tissue Perfluorocarbon Magnetic Resonance Imaging in Mice
Source: NMR Biomed. 2024 Dec 8;38(1):e5296. doi: 10.1002/nbm.5296 (PMC11625660; doi:10.1002/nbm.5296)
Supplement: Supplementary file 1 — Figure S1. Birdcage coil schematic diagram with relevant dimensions (A), labelled diagram of inductive coupling loop (B), close‐up photos of the matching circuitry front (C) and back (D). Figure S2. B1 + maps acquired for 1H excitation with the single‐frequency 1H birdcage coil and the dual‐frequency 1H/19F surface coil using the Bloch–Siegert Shift method with a flip angle of 30°. The 50 mL 0.5% agarose phantom doped with 200 μL of Gd‐DTPA, axial (B) and coronal (C) slices of the B1 + map and corresponding magnitude image for the single‐frequency 1H birdcage coil, and axial (D) and sagittal (E) slices of the B1 + map and corresponding magnitude image for the dual‐frequency 1H/19F surface coil. Figure S3. NMR analysis of six samples of decreasing 19F concentrations used for in vitro sensitivity measurements. Large TFA reference peak and CS‐1000 sample resonance peaks are labelled. Figure S4. Additional photos of in vivo imaging setup on the General Electric Healthcare Discovery MR750 3.0T™ clinical MR scanner. View of birdcage coil and animal cradle on scanner bed during experiment set up (A), imaging set up inside the bore of the MR scanner (B), animal heating and temperature control module held within the control room (C). Figure S5. Full survey of 19F SNR measurements in accessory axillary, sciatic, popliteal, renal, subiliac and lumbar lymph nodes, showing changes in individual SNR measurements due to differences in coil performance. Figure S6. Coronal 19F MR images of PFC localization in the livers of mice, showing differences in bSSFP banding from each of the three 19F compatible coils (dual‐frequency surface coil, dual‐frequency birdcage coil and single‐frequency birdcage coil). Line profiles showing SNR as a function of vertical position from analysis of a vertical cut (indicated by the dotted yellow line). [file NBM-38-e5296-s001.docx]

Supporting Information

**Single-Frequency Birdcage Coils for Deep Tissue Perfluorocarbon Magnetic Resonance Imaging in Mice**

**Sean W. McRae ^1^**, Francisco M. Martinez ^1^, Paula J. Foster ^1^, John A. Ronald ^1,2,3#^, Timothy J. Scholl ^1,2 ,4,5#*^

^1^Department of Medical Biophysics, University of Western Ontario, London, ON, Canada

^2^Imaging Laboratories, Robarts Research Institute, University of Western Ontario, London, ON, Canada

^3^Lawson Health Research Institute, London, ON, Canada

^4^Department of Physics and Astronomy, ON, Canada

^5^Ontario Institute for Cancer Research, Toronto, ON, Canada

* Correspondence can be addressed to T.J.S. (scholl@uwo.ca)

**^#^** Indicates co-senior authors

**CONTENTS**

**Supplemental Figure 1:** Birdcage coil schematic diagram with relevant dimensions (A), labeled diagram of inductive coupling loop (B), close-up photos of the matching circuitry front (C) and back (D).

**Supplemental Figure 2:** B_1_^+^ maps acquired for ^1^H excitation with the single frequency ^1^H birdcage coil and the dual frequency ^1^H/^19^F surface coil using the Bloch-Siegert Shift method with a flip angle of 30 degrees. The 50 mL 0.5% agarose phantom doped with 200 $\mu$L of Gd-DTPA, axial (B) and coronal (C) slices of the B_1_^+^ map and corresponding magnitude image for the single frequency ^1^H birdcage coil, and axial (D) and sagittal (E) slices of the B_1_^+^ map and corresponding magnitude image for the dual frequency ^1^H/^19^F surface coil.

**Supplemental Figure 3:** NMR analysis of six samples of decreasing ^19^F concentrations used for *in vitro* sensitivity measurements. Large TFA reference peak and CS-1000 sample resonance peaks are labeled.

**Supplemental Figure 4:** Additional photos of *in vivo* imaging setup on the General Electric Healthcare Discovery MR750 3.0T™ clinical MR scanner. View of birdcage coil and animal cradle on scanner bed during experiment set up (A), imaging set up inside the bore of the MR scanner, animal heating and temperature control module held within the control room (C).

**Supplemental Figure 5:** Full survey of ^19^F SNR measurements in accessory axillary, sciatic, popliteal, renal, subiliac, and lumbar lymph nodes, showing changes in individual SNR measurements due to differences in coil performance.

**Supplementary Figure 6:** Coronal ^19^F MR images of PFC localization in the livers of mice, showing differences in SSFP banding from each of the three ^19^F compatible coils (dual-frequency surface coil, dual-frequency birdcage coil, and single-frequency birdcage coil). Line profiles showing SNR as a function of vertical position from analysis of a vertical cut (indicated by the dotted yellow line).


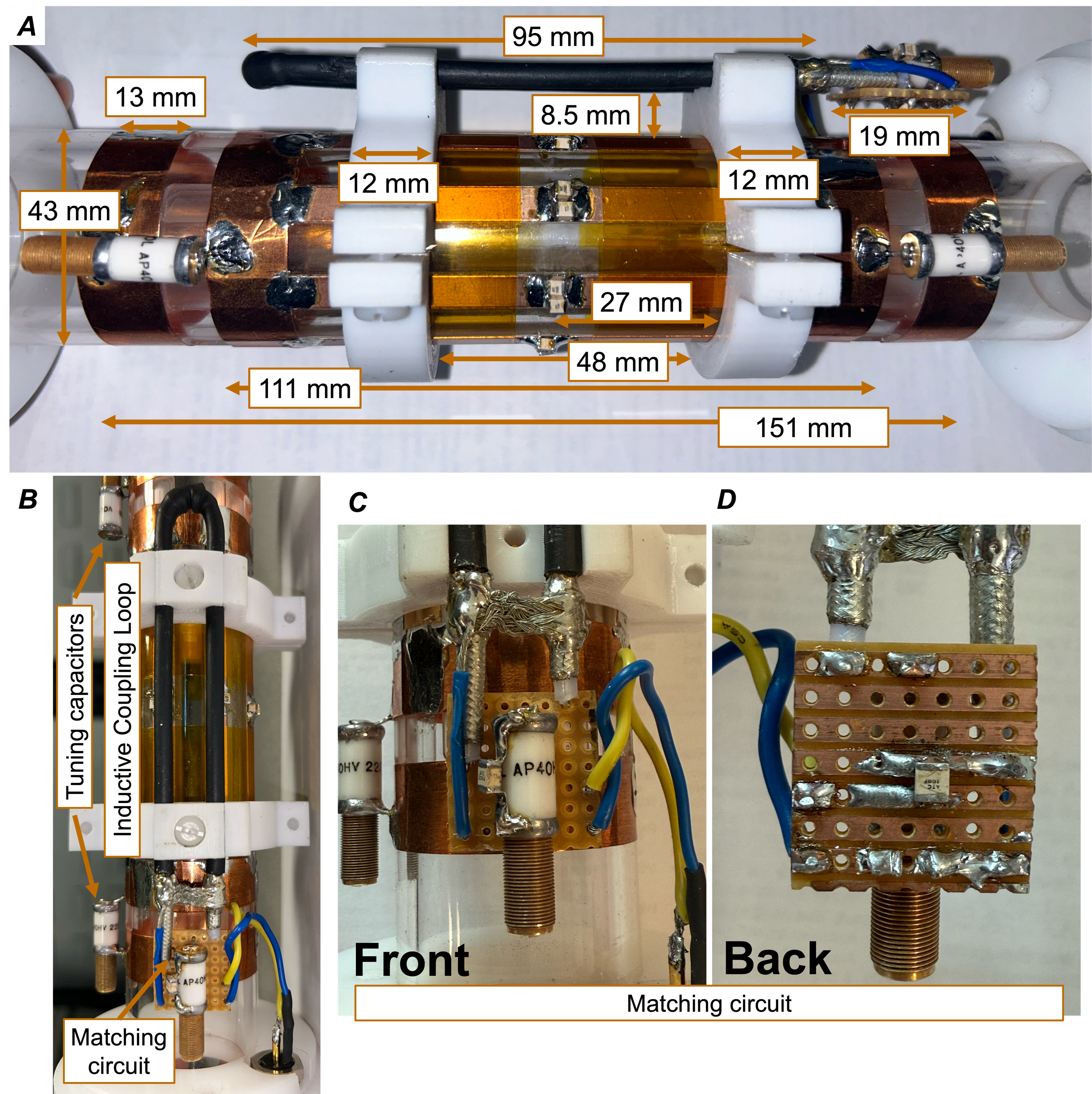
 **Supplemental Figure 1:** Birdcage coil schematic diagram with relevant dimensions (A), labeled diagram of inductive coupling loop (B), close-up photos of the matching circuitry front (C) and back (D).

**
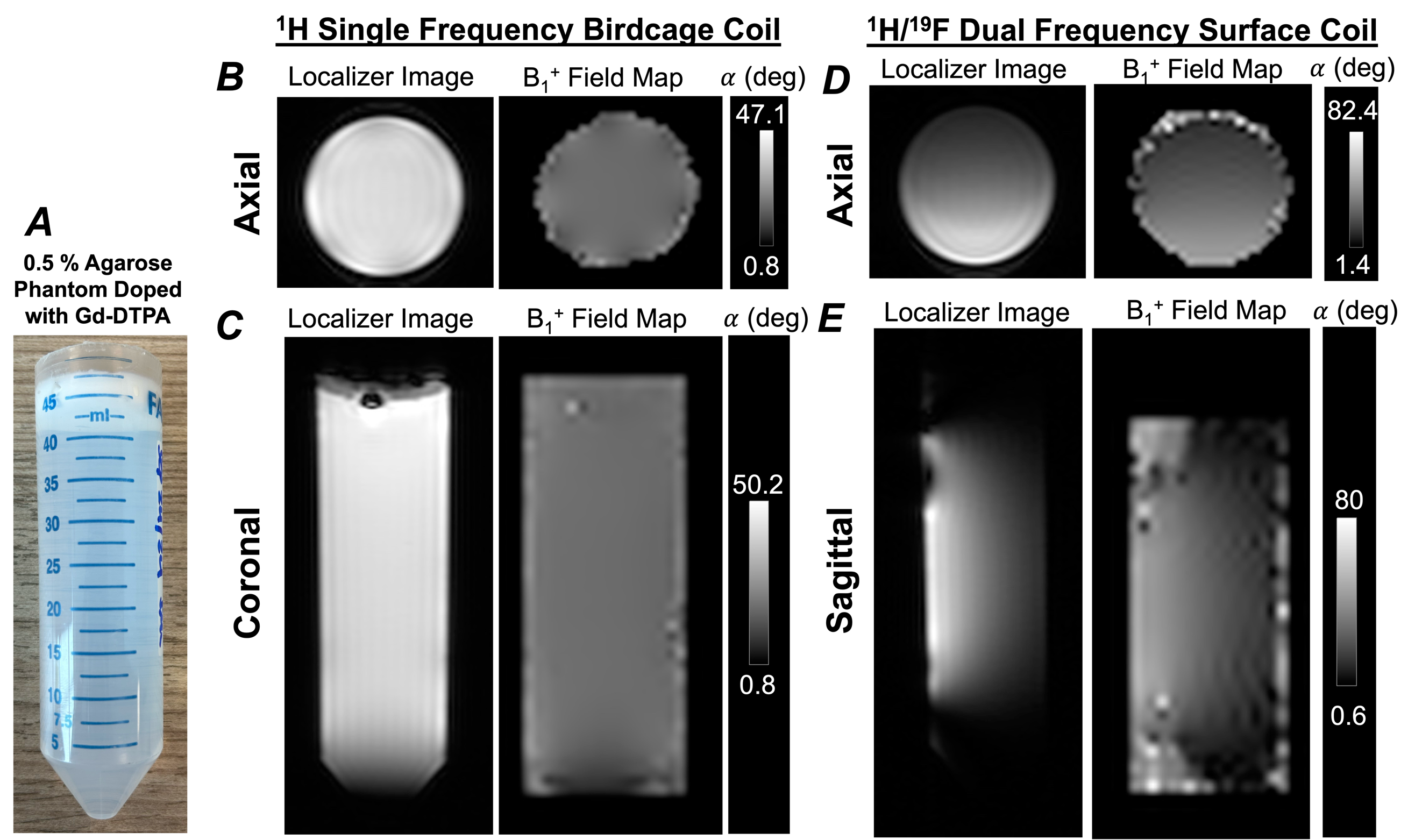
**

**Supplemental Figure 2:** B_1_^+^ maps acquired for ^1^H excitation with the single frequency ^1^H birdcage coil and the dual frequency ^1^H/^19^F surface coil using the Bloch-Siegert Shift method with a flip angle of 30 degrees. The 50 mL 0.5% agarose phantom doped with 200 $\mu$L of Gd-DTPA, axial (B) and coronal (C) slices of the B_1_^+^ map and corresponding magnitude image for the single frequency ^1^H birdcage coil, and axial (D) and sagittal (E) slices of the B_1_^+^ map and corresponding magnitude image for the dual frequency ^1^H/^19^F surface coil.


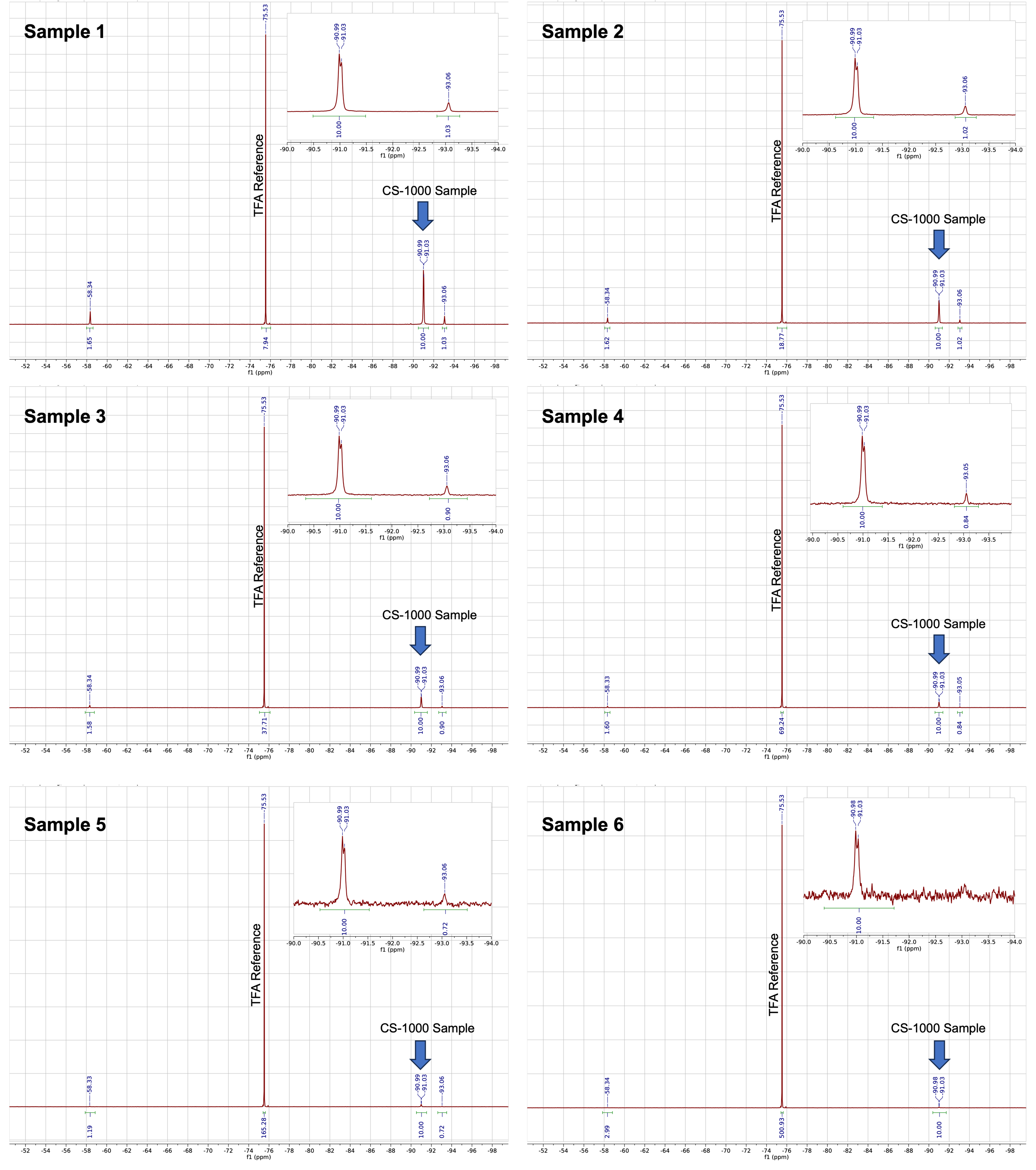
**Supplemental Figure 3:** NMR analysis of six samples of decreasing ^19^F concentrations used for *in vitro* sensitivity measurements. Large TFA reference peak and CS-1000 sample resonance peaks are labeled.


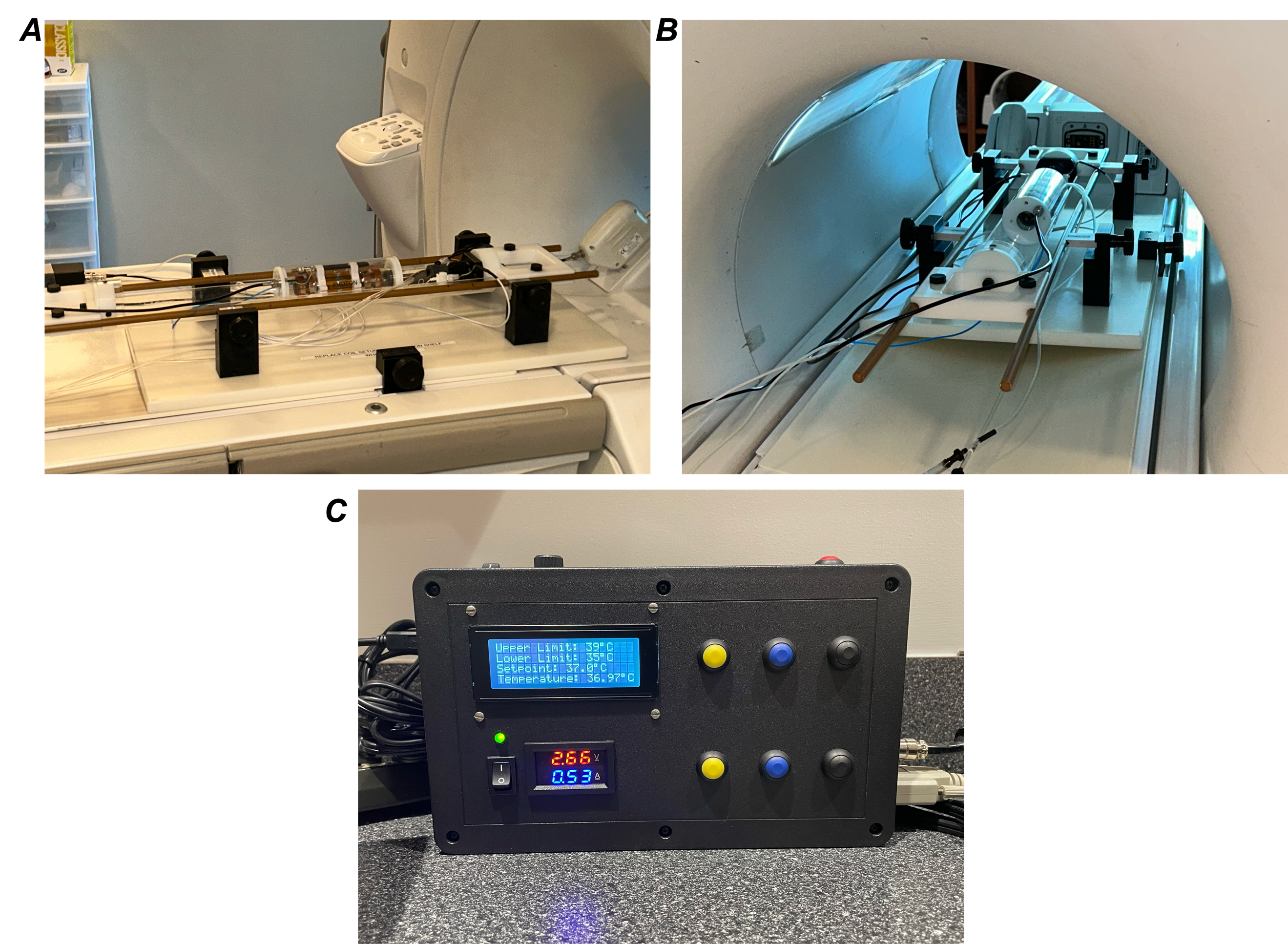


**Supplemental Figure 4:** Additional photos of *in vivo* imaging setup on the General Electric Healthcare Discovery MR750 3.0T™ clinical MR scanner. View of birdcage coil and animal cradle on scanner bed during experiment set up (A), imaging set up inside the bore of the MR scanner (B), animal heating and temperature control module held within the control room (C).

**
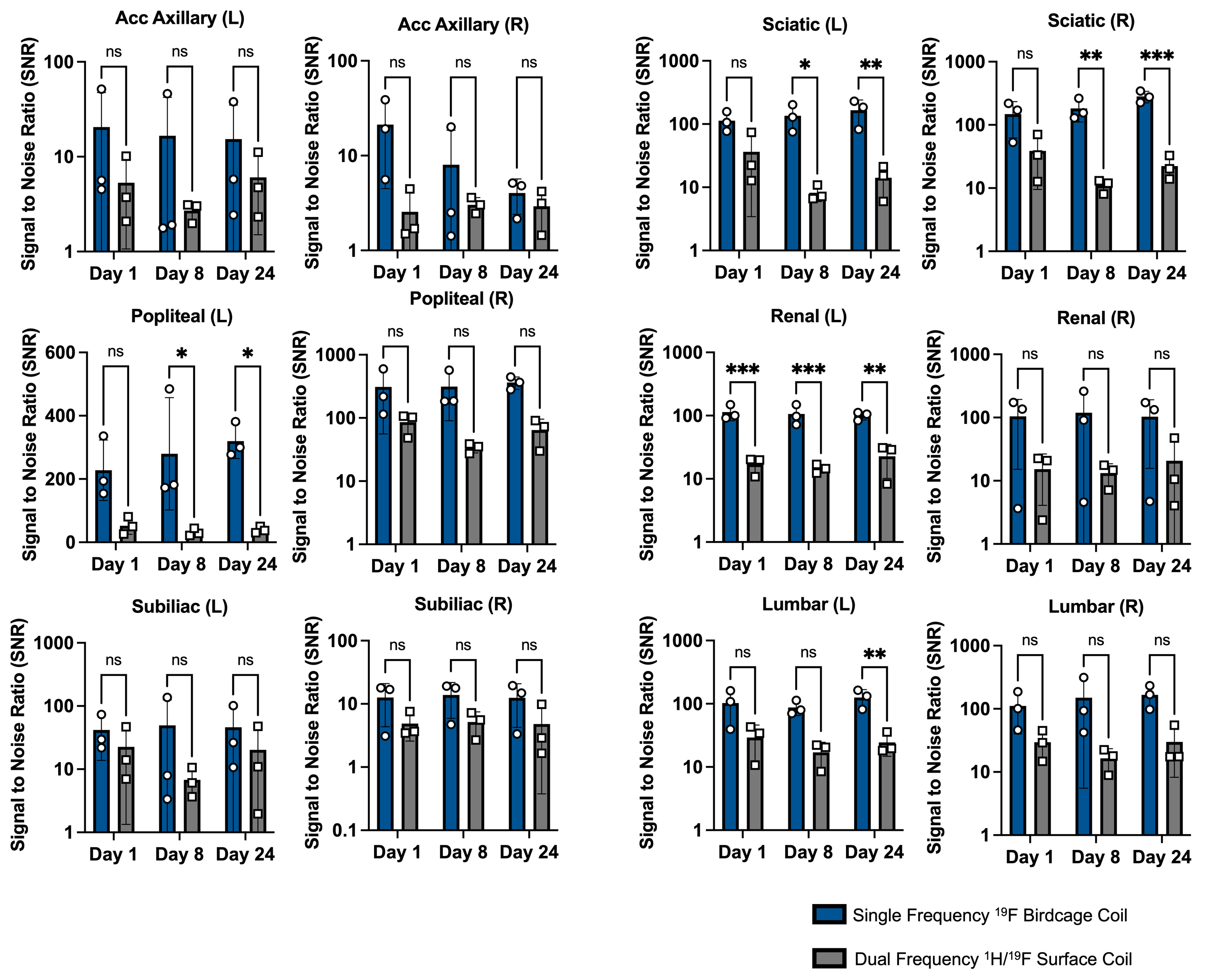
Supplemental Figure 5:** Full survey of ^19^F SNR measurements in accessory axillary, sciatic, popliteal, renal, subiliac, and lumbar lymph nodes, showing changes in individual SNR measurements due to differences in coil performance.

**
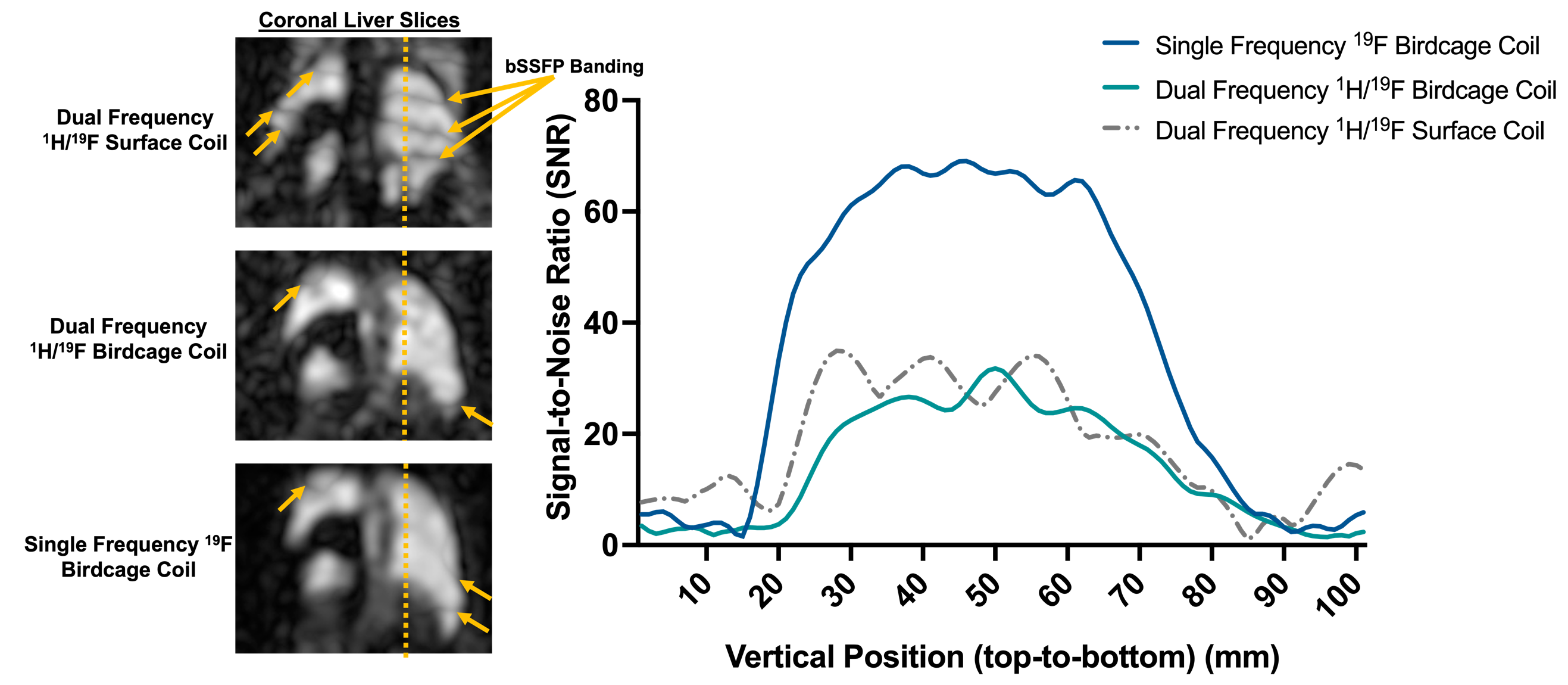
Supplementary Figure 6:** Coronal ^19^F MR images of PFC localization in the livers of mice, showing differences in bSSFP banding from each of the three ^19^F compatible coils (dual-frequency surface coil, dual-frequency birdcage coil, and single-frequency birdcage coil). Line profiles showing SNR as a function of vertical position from analysis of a vertical cut (indicated by the dotted yellow line).
